# Real-World Experience Measurement of Liver Iron Concentration by R2 vs. R2 Star MRI in Hemoglobinopathies

**DOI:** 10.3390/diagnostics10100768

**Published:** 2020-09-29

**Authors:** Riad Abou Zahr, Barbara E. U. Burkhardt, Lubaina Ehsan, Amanda Potersnak, Gerald Greil, Jeanne Dillenbeck, Zora Rogers, Tarique Hussain

**Affiliations:** 1 Pediatric Cardiology Division, Department of Pediatrics, UT Southwestern Medical Center, Dallas, TX 75235, USA; Amanda.Potersnak@childrens.com (A.P.); Gerald.Greil@utsouthwestern.edu (G.G.); mohammad.hussain@utsouthwestern.edu (T.H.); 2 Department of Surgery, Pediatric Cardiology, Pediatric Heart Center, University Children’s Hospital Zurich, 8032 Zurich, Switzerland; Barbara.Burkhardt@kispi.uzh.ch; 3 Department of Pediatrics, University of Virginia School of Medicine, Charlottesville, VA 22908, USA; lubaina.ehsan@gmail.com; 4 Department of Radiology, UT Southwestern Medical Center, Dallas, TX 75235, USA; JEANNE.DILLENBECK@childrens.com; 5 Pediatric Hematology Oncology Division, Department of Pediatrics, UT Southwestern Medical Center, Dallas, TX 75235, USA; Zora.rogers@utsouthwestern.edu

**Keywords:** liver iron concentration, MRI, R2 star, T2 star, R2

## Abstract

Background: Non-invasive determination of liver iron concentration (LIC) is a valuable tool that guides iron chelation therapy in transfusion-dependent patients. Multiple methods have been utilized to measure LIC by MRI. The purpose of this study was to compare free breathing R2* (1/T2*) to whole-liver Ferriscan R2 method for estimation of LIC in a pediatric and young adult population who predominantly have hemoglobinopathies. Methods: Clinical liver and cardiac MRI scans from April 2016 to May 2018 on a Phillips 1.5 T scanner were reviewed. Free breathing T2 and T2* weighted images were acquired on each patient. For T2, multi-slice spin echo sequences were obtained. For T2*, a single mid-liver slice fast gradient echo was performed starting at 0.6 ms with 1.2 ms increments with signal averaging. R2 measurements were performed by Ferriscan analysis. R2* measurements were performed by quantitative T2* map analysis. Results: 107 patients underwent liver scans with the following diagnoses: 76 sickle cell anemia, 20 Thalassemia, 9 malignancies and 2 Blackfan Diamond anemia. Mean age was 12.5 ± 4.5 years. Average scan time for R2 sequences was 10 min, while R2* sequence time was 20 s. R2* estimation of LIC correlated closely with R2 with a correlation coefficient of 0.94. Agreement was strongest for LIC < 15 mg Fe/g dry weight. Overall bias from Bland–Altman plot was 0.66 with a standard deviation of 2.8 and 95% limits of agreement −4.8 to 6.1. Conclusion: LIC estimation by R2* correlates well with R2-Ferriscan in the pediatric age group. Due to the very short scan time of R2*, it allows imaging without sedation or anesthesia. Cardiac involvement was uncommon in this cohort.

## 1. Introduction

Chronic iron overload affects predominantly the liver, pancreas and heart, leading to systemic complications, including liver fibrosis, diabetes and cardiomyopathy [1,2]. Studies have shown that liver iron concentration (LIC) is directly proportional to total body iron stores [3]. Thus, assessment of LIC is indicated prior to initiation of chelation therapy and during therapy to guide management in chronically transfused patients [4]. Historically, LIC was measured via liver biopsy [5]. Due to the invasive nature of liver biopsies and to their high sampling variability, non-invasive alternatives emerged, such as magnetic resonance imaging (MRI), biomagnetic susceptometry and computed tomography (CT) [6,7]. MRI has been the most studied modality.

Measurement of cardiac and liver iron concentration (LIC) by MRI is accurate and reproducible, and has become the test of choice [8,9]. Several techniques have been developed to measure iron by MRI [7,10,11]. One of the well-established methods utilizing the transverse relaxation time constant (T2) is Ferriscan analysis (Resonance Health, Australia). This is based on voxel summations of mean transverse relaxation rates R2, the reciprocal of T2, which is proportional to LIC in a curvilinear relation [12]. Ferriscan analysis has been calibrated against liver biopsy and has proven to be consistent across different MRI vendors [13]. Another commonly used method for quantifying liver and cardiac iron by MRI is via the transverse relaxation time rate R2* (=1/T2*). R2* is proportional to LIC in a linear relation [14]. It may show some advantages in terms of speed (and hence cost) and the ability to perform imaging, even in very young children without the need for anesthesia.

In many institutions, R2 Ferriscan has been the standard method to measure LIC. These two methods have been previously compared in children employing breath holding and multiple slice acquisition for T2* [15]. In this report, we describe our real-world experience where we compare simultaneous LIC derived from R2 (Ferriscan) to that derived from free breathing single slice R2* in a cohort of pediatric and young adult patients who predominantly have hemoglobinopathies receiving chronic transfusion therapy.

## 2. Materials and Methods

### 2.1. MRI

We conducted a single institution retrospective observational study on liver iron MRIs from April 2016 to May 2018. The study was approved by the local ethics committee (IRB STU 032016-009 10 June 2016) for retrospective analysis of MRI and clinical data, with a waiver of individual patient consent. Clinically indicated liver iron MRI studies were performed on a 1.5 T scanner (Ingenia, Philips Healthcare, Best, The Netherlands) using a 32-channel torso coil. Free breathing T2 and T2* weighted images were acquired on each patient. For T2, transverse images were obtained via a multi-slice spin echo sequence with repetition time (TR) of 1000 ms over five acquisitions with echo times of 6, 9, 12, 15 and 18 ms. To cover the whole liver, 11 slices were acquired with a flip angle of 90 degrees, slice thickness of 6 mm, and gap of 6 mm. A saline bag (1L) was positioned over the patient’s left side to correct for signal changes. For T2*, transverse images were acquired via a single mid-hepatic slice fast gradient echo sequence with TR 13–25 ms and 10 echo times starting at 0.6 ms with 1.2 ms increments with 8 signal averages. The slice thickness was 20 mm, flip angle 30 degrees and in-plane resolution 2 × 2 mm. For occasional patients with T2* decay from qualitative viewing of greater than 6 ms, a second sequence was acquired to allow greater sampling of the decay at low iron concentrations. This sequence has 10 echo times starting at 2.3 ms and increases in 2.3 ms increments. There are three signal averages, a slice thickness of 10 mm, in-plane resolution 2 × 2 mm and a flip angle of 30 degrees.

Myocardial T2* imaging was performed per hospital protocol for cases with severe iron overload. Images were acquired using a single short axis diastolic mid-ventricular slice fast gradient echo sequence with eight echoes starting at 2.6 ms with 2.6 ms increment with electrocardiogram (ECG) gating and breath holding when possible, or otherwise signal averages. The slice thickness was 10 mm, repetition time 21 ms, flip angle 30 degrees and in-plane resolution 1.2 × 1.2 mm with a black blood preparation pre-pulse.

### 2.2. Analysis

T2 images were commercially analyzed off-site at Resonance Health using Ferriscan analysis with a turnaround time of 2–3 business days. Quantitative T2* maps were calculated offline. T2* gradient echo images were fitted to a mono-exponential decay curve for data validation and noise filtration using Osirix MD software (Pixmeo SARL, Bernex, Switzerland). Subsequent quantitative analysis was performed using cmr42 software (Circle Cardiovascular Imaging Inc., Calgary, AB, Canada). Multiple regions of interest (ROI) were drawn on T2* maps on homogenous regions within the right lobe of the liver parenchyma avoiding major vessels and bile ducts. Mean T2* values were converted to R2* using the formula R2*[Hz] = 1000/T2*[ms]. Then, LIC (in mg/g dry weight) was calculated using the equation: [Fe]R2* = 0.0254 × R2* + 0.202 [14]. Cardiac iron quantification of T2* images was carried out by drawing a single ROI in the mid ventricular septum [16].

Mean ± standard deviation was used to describe continuous variables. Agreement between the two methods was evaluated by linear regression and Bland–Altman analysis using 95% confidence intervals [17]. A scatter plot was generated to demonstrate the relation between R2 and R2* LICs. 

## 3. Results

One hundred and seven patients were included in this study (Table 1). Mean age was 12.5 ± 4.5 years (range 2–24 y). The patients’ primary diagnoses were sickle cell anemia, thalassemia, Blackfan Diamond anemia and malignancies. The most prevalent diagnosis was sickle cell anemia. All liver scans were performed free breathing. Four patients less than 5 years of age required sedation to complete the study. Average scan time for running all five T2 sequences was 10 min, while the average time for running the T2* sequence was 20 s.

**Figure 1:**
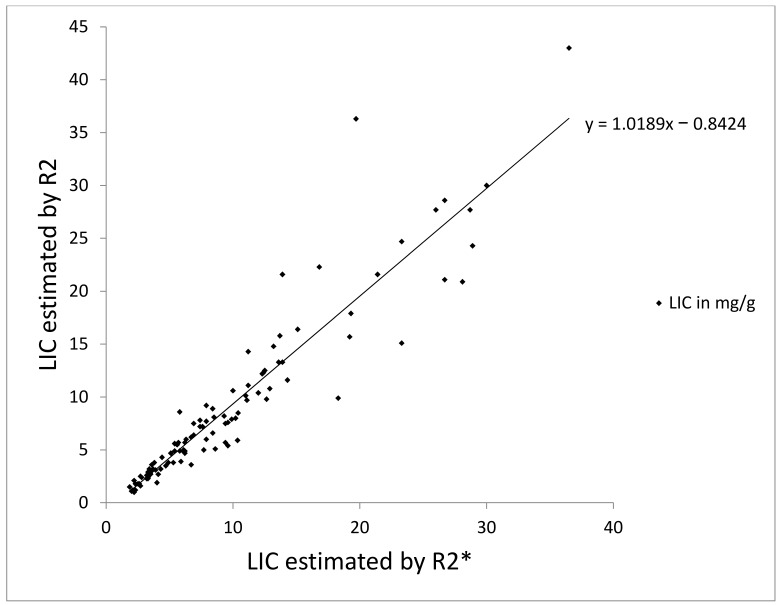
Scatter plot of liver iron concentration (LIC) by R2* versus LIC by R2 with linear regression line. The slope is 1.01 ± 0.03, the intercept is −0.84 ± 0.44 and *r*^2^ is 0.88. The correlation coefficient for R2* and R2 is 0.94.

**Figure 2:**
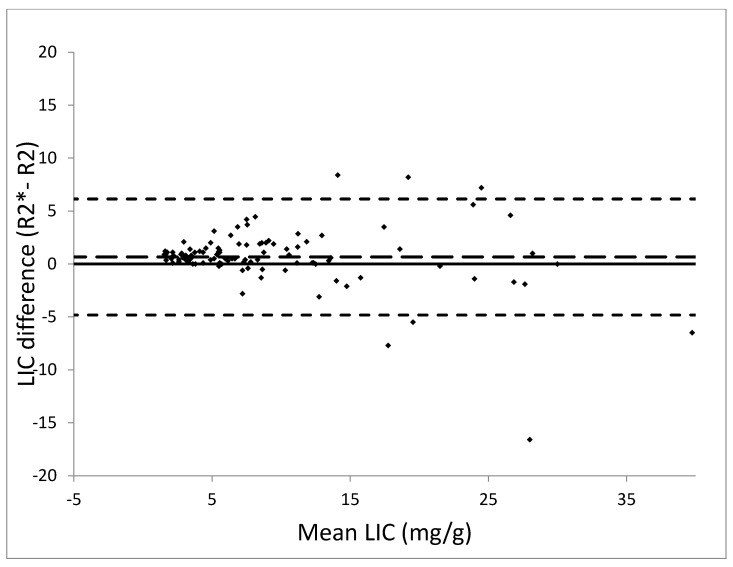
Bland–Altman plot analysis demonstrates strong agreement between LIC R2* and R2 methods. Note scatter increases with increasing LIC. The small-dashed lines indicate the upper and lower 95% limits of agreement between the two measurements. The large-dashed line represents the mean difference.

Mean LIC by R2* was 9.5 ± 7.4 mg Fe/g dry weight of liver (range, 1.9–36.5 mg Fe/g dry weight). Mean LIC by R2 was 8.9 ± 8.1 mg Fe/g dry weight (range, 1–43 mg Fe/g dry weight). LIC by R2* was plotted against LIC by R2 in a linear fit with a regression slope of 1.01 ± 0.03, a y intercept of −0.8 ± 0.4, *r*^2^ = 0.88 and a correlation coefficient of 0.94 (Figure 1). Bland–Altman plot analysis revealed an overall bias between LIC means of 0.66 mg Fe/g dry weight (CI 0.125 to 1.195) with a standard deviation of 2.8, suggesting an overall tendency of R2* for overestimation. The 95% limits of agreement between R2 and R2* LIC were 6.1 (CI 5.228 to 7.052) and −4.8 (CI −5.732 to −3.908) (Figure 2).

Agreement between the two methods was strongest for LIC ≤ 15 mg Fe/g dry weight. More scatter was observed with increasing LIC, specifically LIC > 15 mg Fe/g dry weight. Using LIC ≥ 15 mg Fe/g dry weight as the cutoff for severe iron overload [2,12], R2* LIC had two false negatives (underestimated LIC from severe to moderate) and one false positive (overestimated LIC from moderate to severe). This resulted in a misclassification error of 2.8% (CI 0.3–5.9%) and an accuracy of 97% for severe iron loading. Similarly, using LIC ≥ 7 mg Fe/g dry weight as the cutoff for moderate iron overload [2,14], R2* had two false negatives (underestimated LIC to mild) and six false positives (overestimated LIC to moderate). This generated a misclassification error of 7% (CI 1.9–11.2%) and an accuracy of 93% for moderate iron loading. For the normal LIC < 1.8 mg Fe/g dry weight, R2* had one false negative and four false positives with a misclassification rate of 5% (CI 0.7–8.7%) and an accuracy of 95%.

Twelve (11%) patients with severe liver iron loading underwent cardiac iron imaging and quantification using T2*. Two of these patients had T2* < 10 ms, consistent with severe cardiac iron overload [18].

## 4. Discussion

This study is a real-world experience comparing LIC estimation by free breathing R2* with R2 Ferriscan method in 107 patients who predominantly have hemoglobinopathies on chronic transfusion therapy. It demonstrates excellent agreement of LIC R2* with LIC R2 Ferriscan at clinically relevant LIC levels with low classification error rates. This strong agreement between the two methods has been demonstrated in separate cohorts of thalassemia and sickle cell anemia patients [14,19]. T2* cardiac and liver imaging has been shown to be highly reproducible with low coefficient of variation across different MRI vendors [8]. Furthermore, this study highlights the convenience of R2* in the pediatric age group. T2* imaging can be performed via a single breath hold in a very short period of time. Alternatively, T2* acquisition via a single slice may be performed with free breathing employing signal averaging over 20 s, as was done in our study. This is highly advantageous in children due to their frequent inability to perform appropriate breath holding and to the difficulty in lying supine without movement for several minutes. Thus, it obviates the need for sedation or general anesthesia in most patients.In practice, free breathing R2* is a valid method in children and whenever Ferriscan analysis is not available.

R2 relaxometry analysis by Ferriscan is an FDA approved technique. It has high sensitivity and specificity at clinically important LICs [12]. R2 Ferriscan is commercially available only for 1.5T scanners. This method is less sensitive to the imaging voxel dimensions and more resistant to susceptibility artifacts than R2* techniques [14]. Acquisition time is rather long (at least 10 min), which represents a challenge in the pediatric age group. In children, motion artifact is common and at times necessitates repeated acquisitions and further prolongs total scan time. Data analysis is complex and occurs at a central laboratory with a couple of business days turnaround report time [11]. Furthermore, Ferriscan is a commercial product that charges a fee for every individual scan, which is a financial burden on any institution. On the other hand, R2* data analysis via ROIs is rather simple and is readily available on most post-processing computers without an additional charge. We used multiple small ROIs to sample most of the liver parenchyma. Several groups use 3-4 small ROIs for analysis [20]. Whole-liver ROI has been described for R2* analysis, and in one study was found to be more consistent than small ROI analysis [21]. The observed tendency of R2* for overestimation may perhaps be related to the increased slice thickness (20 mm) that was used to acquire the standard 2D multi-echo fast gradient echo.

Sickle cell and thalassemia patients comprised the majority of our cohort. It has been shown that distribution of hepatic iron is similar in these two diseases and remains comparable with iron chelation [22]. The increasing scatter observed at LIC > 15 mg Fe/g dry weight between R2* and R2 is expected and has been previously demonstrated [15,23]. R2 LIC has a curvilinear relationship when compared to liver biopsy with more scatter at high iron levels. Specifically, for LIC > 15 mg Fe/g dry weight liver, R2 sensitivity for biopsy LIC prediction drops to 85% [12]. In addition, liver biopsy itself has higher sampling variability with severe iron deposition [6,9,24,25,26]. R2*, unlike R2, maintains a linear relation with severe iron overload [27]. All these factors play a role in the apparently reduced accuracy of R2* at high LIC. Hankins et al. compared LIC by breath-hold R2* to liver biopsy in 47 pediatric patients [23]. Similar to our findings, they observed more scatter for LIC > 15 mg Fe/g dry weight liver. This is due to the rapid signal decay of T2* in addition to limitations in the ability to generate a starting ultra-short echo time with standard techniques. The minimum echo time for T2* in our study was 0.6 ms. Lower echo times were not possible using the standard Cartesian k-space acquisition available in our scanner. Thus, high levels of LIC cannot be accurately determined with the current technique. This is a known limitation of T2* technique [15]. Using faster imaging trajectories that employ radial acquisition can allow generation of ultrashort echo times and estimation of higher LIC [28,29]. However, an advantage of T2* technique is that it can be used on 3T scanners with good agreement with liver biopsy [30]. At 3T, R2* values have been shown to be double those generated on 1.5T [31]. This fact renders LIC estimation by R2* more challenging at 3T, especially in the presence of severe iron overload. Therefore, 1.5T remains the field strength of choice for routine liver iron assessment by MRI.

Cardiac involvement in our cohort of children and young adults was uncommon. This is probably due to the large proportion of sickle cell patients and to the relative younger age of this cohort. Chronically transfused thalassemia patients develop myocardial iron deposition many years before sickle cell patients on chronic transfusions [32]. Mid-ventricular septal iron has been shown to correlate well with global left ventricular iron concentration [16]. Severe iron overload can induce cardiomyopathy, which is reversible with aggressive chelation therapy [33,34]. In general, with advanced states of iron overload, transferrin will be saturated and iron will be free in a non-transferrin bound form. This iron form has more toxicity than bound iron and leads to impaired cardiac ventricular function [35]. Thus, quantification of cardiac iron overload has potential direct clinical benefits [36].

One limitation of this study is that no liver biopsies were performed. Although liver biopsy has long been considered the gold standard for measurement of LICs, this concept has been challenged secondary to its high sampling variability [9]. In addition, this study did not measure serum ferritin levels at the time of the MRI scans, which could have provided more insights into the relation between this marker and LIC. Due to the nature of this study, R2 Ferriscan was considered the reference standard. Furthermore, this study did not evaluate serial measurements per patient. It is recommended not to use R2 and R2* methods for LIC estimation interchangeably but rather to be consistent in using the same method to follow LIC trends in the same patient [19,20].

## 5. Conclusions

LIC estimation by R2* correlates well with R2 Ferriscan in the pediatric age group. Some scatter is observed at higher iron levels but with a low R2* classification error rate. The free-breathing R2* method is highly advantageous in children due to the very short scan time, which allows imaging of most young patients without sedation or anesthesia. Cardiac involvement in our cohort of children and young adults was uncommon.

## Figures and Tables

**Table 1:** Demographic data.

Patient Characteristics	Results
Total number of patients	107
Mean age (years)	12.5
Primary Diagnoses	
Sickle cell anemia	76
Thalassemia	20
Blackfan Diamond anemia	2
Acute lymphoblastic leukemia	8
Wilms tumor	1

## Abbreviations

CI	Confidence interval
CMR	Cardiac magnetic resonance
CT	Computed tomography
ECG	electrocardiogram
Fe	iron
LIC	Liver iron concentration
MRI	Magnetic resonance imaging
ROI	Region of interest
T	Tesla
TR	Repetition time
